# Augmented reality-based navigation increases precision of pedicle screw insertion

**DOI:** 10.1186/s13018-020-01690-x

**Published:** 2020-05-14

**Authors:** Cyrill Dennler, Laurenz Jaberg, José Spirig, Christoph Agten, Tobias Götschi, Philipp Fürnstahl, Mazda Farshad

**Affiliations:** 1 Spine Division, University Hospital Balgrist, University of Zürich, Forchstrasse 340, 8008 Zurich, Switzerland; 2 Department of Radiology, University Hospital Balgrist, University of Zürich, Zurich, Switzerland; 3 Laboratory for Biomechanics, University Hospital Balgrist, University of Zürich, Zurich, Switzerland; 4 Computer Assisted Research and Development Group, University Hospital Balgrist, University of Zürich, Zurich, Switzerland

## Abstract

**Background:**

Precise insertion of pedicle screws is important to avoid injury to closely adjacent neurovascular structures. The standard method for the insertion of pedicle screws is based on anatomical landmarks (free-hand technique). Head-mounted augmented reality (AR) devices can be used to guide instrumentation and implant placement in spinal surgery. This study evaluates the feasibility and precision of AR technology to improve precision of pedicle screw insertion compared to the current standard technique.

**Methods:**

Two board-certified orthopedic surgeons specialized in spine surgery and two novice surgeons were each instructed to drill pilot holes for 40 pedicle screws in eighty lumbar vertebra sawbones models in an agar-based gel. One hundred and sixty pedicles were randomized into two groups: the standard free-hand technique (FH) and augmented reality technique (AR). A 3D model of the vertebral body was superimposed over the AR headset. Half of the pedicles were drilled using the FH method, and the other half using the AR method.

**Results:**

The average minimal distance of the drill axis to the pedicle wall (MAPW) was similar in both groups for expert surgeons (FH 4.8 ± 1.0 mm vs. AR 5.0 ± 1.4 mm, *p* = 0.389) but for novice surgeons (FH 3.4 mm ± 1.8 mm, AR 4.2 ± 1.8 mm, *p* = 0.044).

Expert surgeons showed 0 primary drill pedicle perforations (PDPP) in both the FH and AR groups. Novices showed 3 (7.5%) PDPP in the FH group and one perforation (2.5%) in the AR group, respectively (*p* > 0.005).

Experts showed no statistically significant difference in average secondary screw pedicle perforations (SSPP) between the AR and the FH set 6-, 7-, and 8-mm screws (*p* > 0.05). Novices showed significant differences of SSPP between most groups: 6-mm screws, 18 (45%) vs. 7 (17.5%), *p* = 0.006; 7-mm screws, 20 (50%) vs. 10 (25%), *p* = 0.013; and 8-mm screws, 22 (55%) vs. 15 (37.5%), *p* = 0.053, in the FH and AR group, respectively. In novices, the average optimal medio-lateral convergent angle (oMLCA) was 3.23° (STD 4.90) and 0.62° (STD 4.56) for the FH and AR set screws (*p* = 0.017), respectively. Novices drilled with a higher precision with respect to the cranio-caudal inclination angle (CCIA) category (*p* = 0.04) with AR.

**Conclusion:**

In this study, the additional anatomical information provided by the AR headset superimposed to real-world anatomy improved the precision of drilling pilot holes for pedicle screws in a laboratory setting and decreases the effect of surgeon’s experience. Further technical development and validations studies are currently being performed to investigate potential clinical benefits of the herein described AR-based navigation approach.

## Introduction

Precise insertion of pedicle screws for spinal instrumentation is paramount to achieve primary stability in fusion surgery and to avoid possibly catastrophic complications including permanent nerve or vascular injury. Anatomic landmarks supported by fluoroscopic image guidance are utilized to determine safe pedicle screw trajectories without endangering closely adjacent neurovascular structures. The reported accuracy of pedicle screw placement varies considerably depending on the applied technique of screw placement and patient-specific factors including spinal deformity. Image-based intraoperative techniques such as 2D and 3D fluoroscopy or CT-based navigation increase the precision of pedicle screw placement but significantly increase radiation exposure to the patient and operating room personnel [1–7]. Other promising methods of intraoperative navigation techniques including mechanical drilling aids or CAD-designed and 3D-printed patient-specific instruments are cost intense and may require prolonged preoperative preparation and planning [8–10].

**Fig. 1:**
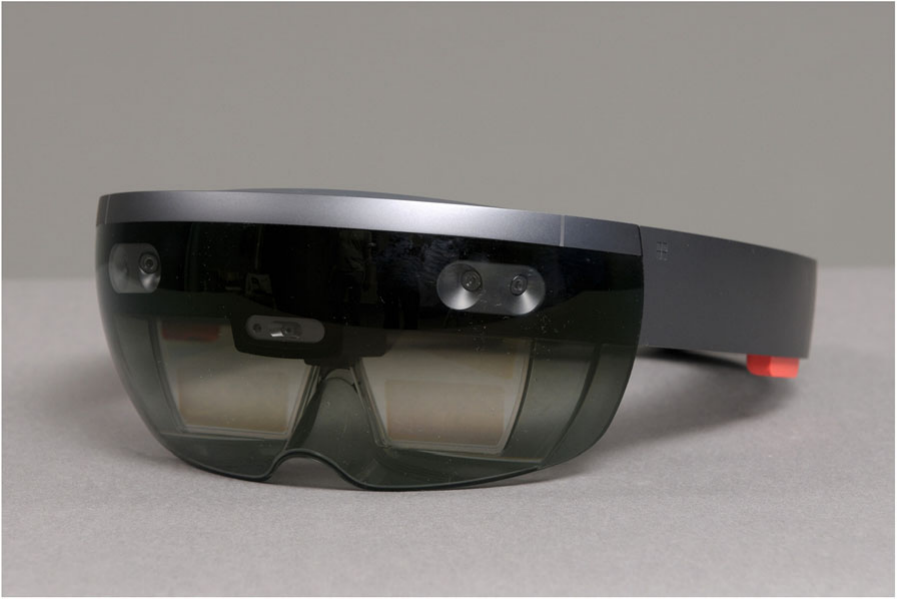
AR headset: Microsoft Hololens (Microsoft, Redmond, WA, USA)

Augmented reality (AR) is a rapidly emerging technology providing the user with computer-generated information superimposed to real-world environment. Although its application in orthopedic and spine surgery today remains limited, AR was gradually introduced in different experimental medical and surgical settings [11–15]. Advancements in information technology and hardware manufacturing transformed former bulky and cable-bound AR headsets into ergonomic devices fulfilling strict requirements of ergonomic design [16]. Recent studies demonstrated that AR may improve accuracy, safety, and efficacy of surgical procedures [17–19]. The aim of this study was to compare the classical free-hand technique of pedicle screw placement to a novel, AR-supported technique using a commercially available state-of-the-art AR headset (Microsoft Hololens®, Microsoft, Redmond, WA, USA) (Fig. 1). The hypothesis of this study was that additional holographic anatomical information provided to the surgeon results in increased precision of setting pilot holes for pedicle screws and compensates for the effect of surgeon’s experience as a confounding factor.

## Materials and methods

**Fig. 2:**
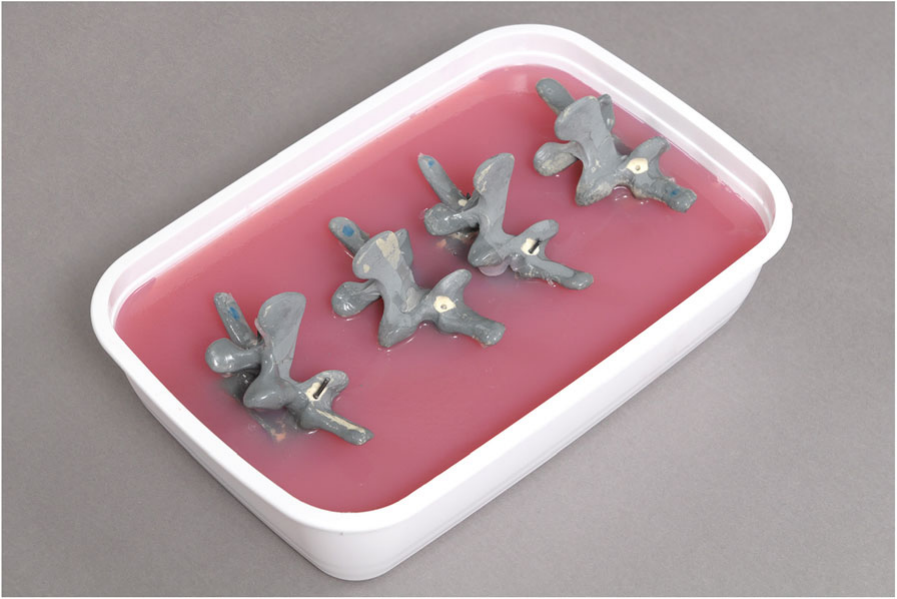
Third lumbar vertebra sawbone models embedded in Repligel (Swiss-Composite, Fraubrunnen, Switzerland)

Eighty identical sawbone models of a third lumbar spinal vertebra (Sawbones Europe AB, Malmö, Sweden; article number: SKU #1375-26-3) were embedded in an agar-based gel (Repligel PG, Swiss-Composite, Fraubrunnen, Switzerland) mimicking exposure of the surgical field in lumbar fusion surgery through a posterior approach. The vertebrae were embedded in different orientations by changing their angulation and rotation by approximately ± 15° in a random direction (Fig. 2). Eighty left and eighty right pedicles were equally randomized into a free-hand (FH) and an augmented reality-supported group (AR).

**Fig. 3:**
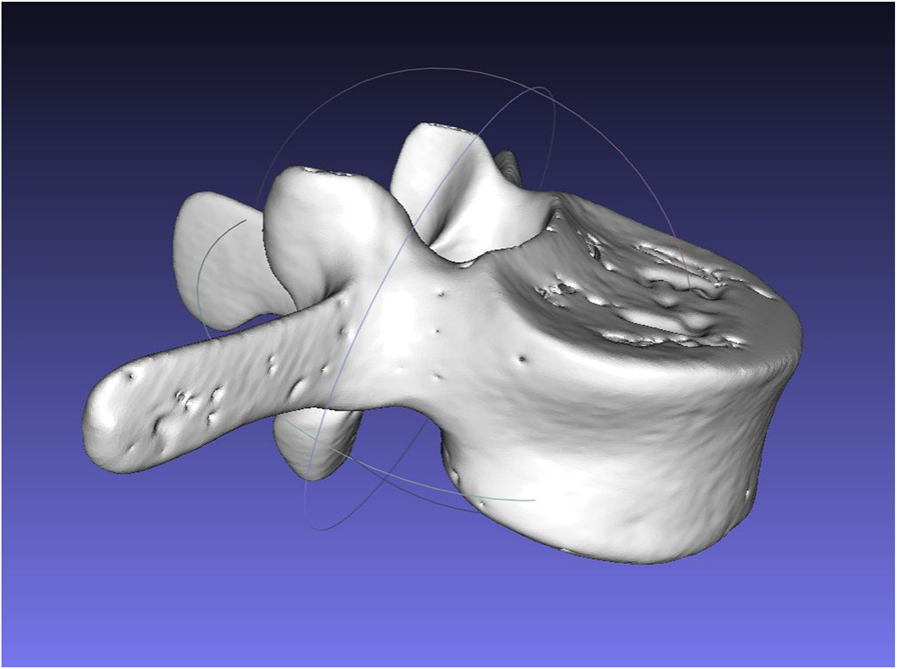
3D model of a single third lumbar sawbone vertebra

**Fig. 4:**
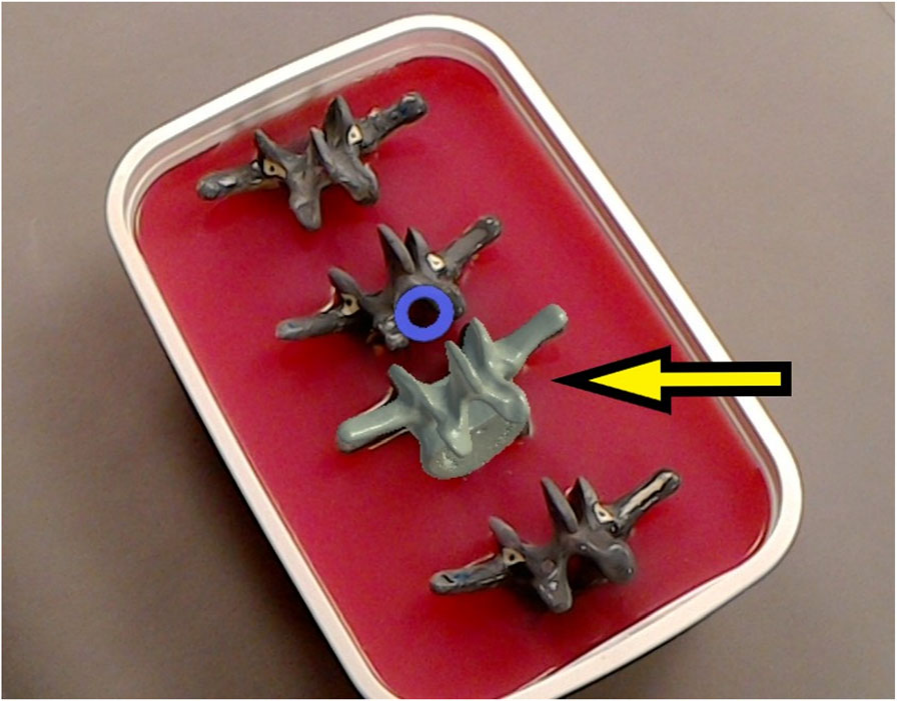
Embedded sawbone vertebrae viewed from the surgeon perspective. The third vertebra (from top) is overlaid by a 3D model of the vertebral body (yellow arrow)

**Fig. 5:**
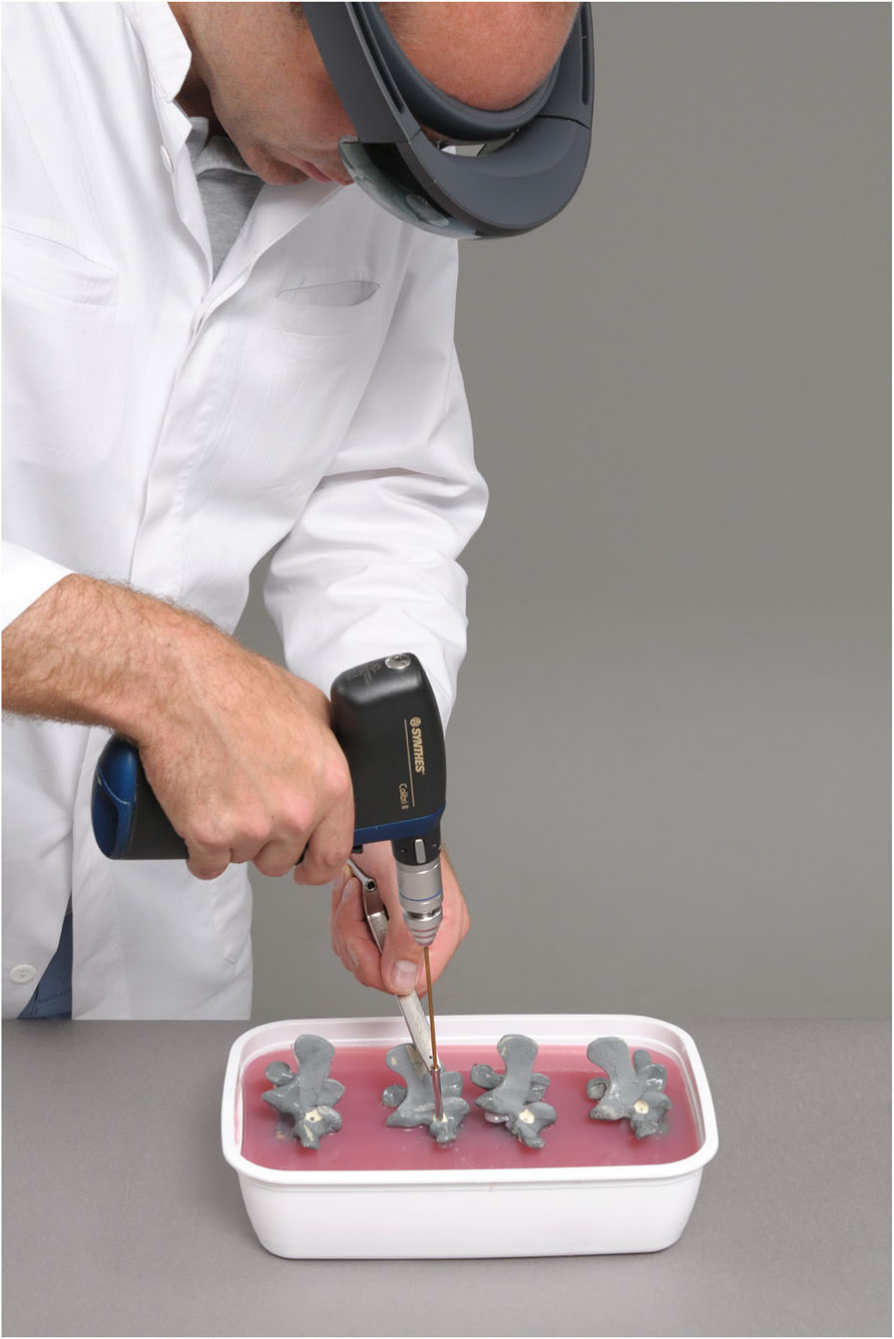
Surgeon drilling a pilot hole with AR navigation

For the AR method, computed tomography (CT) scans (Philips Brilliance 64, Philips Healthcare, Best, The Netherlands) of the vertebral sawbone models were acquired and a 3D triangular surface model was generated using a commercially available software (Siemens syngo.via Frontier 3D printing V 1.0.0, Siemens Healthineers, Erlangen, Germany) (Fig. 3). The 3D model was edited with the Unity software package (version 5.5, Unity Technologies, San Francisco, CA, USA) using threshold segmentation and definition of regions of interest. A proprietary application for the Microsoft Hololens (Microsoft, Redmond, WA, USA) was implemented using Microsoft Visual Studio 2015 (Microsoft, Redmond, WA, USA) permitting interactive rotation and translation of the 3D model by voice commands and gestures. The application was uploaded to the AR headset. Two board-certified orthopedic surgeons specialized in spine surgery and two novice surgeons were each instructed to drill pilot holes for 40 pedicle screws without preoperatively planned trajectories. The pilot holes required to be convergent, matching the angle of the pedicles, drilled as centered as possible to avoid penetration of the pedicle wall and parallel to the vertebral endplate. Before drilling, surgeons were permitted to study the acquired CT scan of the vertebra without performing further measurements. The FH holes were drilled first. For the AR method, the surgeons were supplied with the AR headset providing an overlay between the 3D vertebra model and each embedded sawbone vertebra. The overlay was achieved by translating and rotating the 3D model using voice commands and hand gestures. With the 3D overlay, the entire vertebra body in correct orientation became visible to the surgeon (Figs. 4 and 5).

After drilling, CT scans of the vertebrae were acquired. The pilot holes of each vertebra were marked with pencil leads (Caran d’Ache, Geneva, Switzerland) to facilitate identification of the trajectories in the CT scan.

The following measures were used to assess the quality of pedicle screw placement in the multi-planar reconstructions of the CT scans using standard PACS software (Phönix-PACS GmbH, Freiburg, Germany). The minimal distance of the drill axis to the pedicle wall (MAPW) was defined as the shortest distance between the drill axis and the pedicle wall that could be measured along the entire trajectory. Larger MAPW values correspond to a more centric drill, which allows to use a bigger screw diameter without perforation of the pedicle cortex.

Two measures, namely the number of primary drill pedicle perforations (PDPP) and secondary screw pedicle perforations (SSPP), were introduced to quantify drill perforation of the pedicle wall. The PDPP measure was defined as the perforation of the drill axis itself, represented by a two-dimensional (2D) line. Three SSPP measures were defined by cylinders having outer diameters equal to 6-, 7-, and 8-mm pedicle screws, respectively.

The optimal medio-lateral convergent angle (oMLCA) was defined as the angle between the sagittal plane of the vertebral body and the pilot hole that would result in the best centricity of a screw within the pedicle. In case of our vertebra model, the angle was measured and calculated to be 15°. Based on the oMLCA and the measured true medio-lateral convergence angle (tMLCA) of the pilot hole, the deviation of the optimal medio-lateral convergence angle (doMLCA) was calculated as the difference between tMLCA and oMLCA. Positive values correlate with greater convergence of the pilot hole.

The cranio-caudal inclination angle (CCIA) of a pilot hole was defined as the angle between the endplate (i.e., sagittal plane) and the pilot hole. A CCIA of 0° parallel to the endplate was considered to be optimal. Positive CCIA correlated with a more caudal direction of the screw. More caudally directed screws were rated acceptable and not considered as a surgical failure, because only deviation towards the endplate are associated with increased risk of endplate perforation and early screw fatigue or failure [20]. With respect to the CCIA, we categorized screws in “high-,” “medium-,” and “low-precision” screws. CCIA values between − 2.5 and + 10° denote high-precision, CCIA values between − 2.5 and −5° or between + 10 and + 20° denote medium-precision, and CCIA values below − 5 or above 20° represented low-precision screws.

### Statistical analysis

Statistical analysis was conducted with SPSS (IBM SPSS Statistics for Windows, version 24.0. Armonk, NY). To measure the effect of the navigation method and the skill level of a surgeon on MAPW, independent samples *t* tests were used for each skill level separately to compare FH vs. AR navigation. The interaction term of navigation method and surgeon’s level of experience was added to the model to detect potentially opposite effects of the navigation technique depending on the level of experience of the surgeon. The number of screw perforations for different screw diameters depending on the navigation method was analyzed for both levels of experience separately using Fisher’s exact test. The deviation of the orientation between placed screw and planned screw depending on the navigation technique was compared for both levels of experience separately with independent samples *t* tests. Level of significance was set at *p* < 0.05.

## Results

**Fig. 6:**
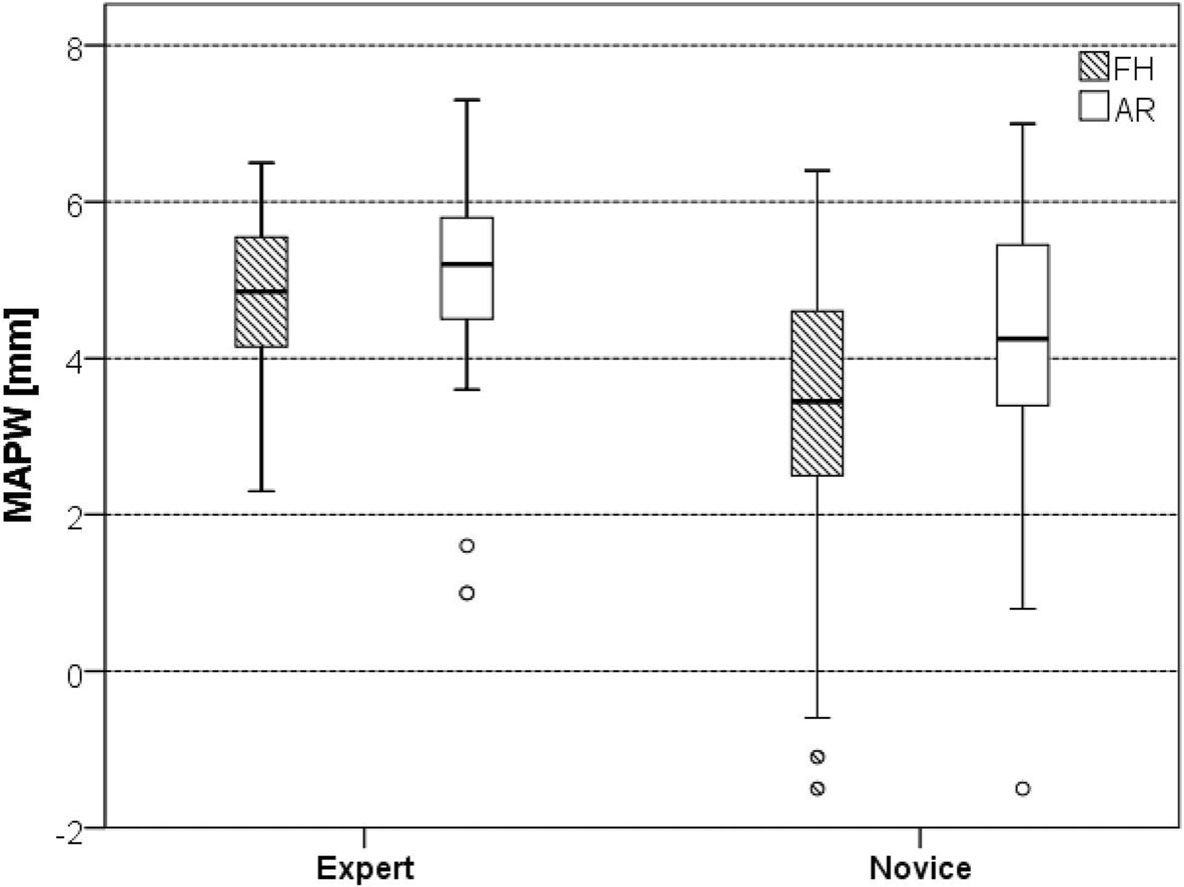
Minimal axis-pedicle wall distance (MAPW)

The average MAPW was similar for both techniques in the hand of the expert surgeons (FH 4.8 ± 1.0 mm vs. AR 5.0 ± 1.4 mm, *p* = 0.389) but higher in the screw trajectories set by the novice surgeons (FH 3.4 mm ± 1.8 mm, AR 4.2 ± 1.8 mm, *p* = 0.044) (Fig. 6). This reflects a better centering of the pilot hole axes in the AR group compared to the FH group for novices.

Expert surgeons showed a PDPP with 0 perforations in both the FH and AR groups. Novices showed a PDPP of 3 perforations (7.5%) in the FH group and one perforation (2.5%) in the AR group, respectively. There was no significant difference between FH and AR navigation for both experts and novices (Table 1).

**Table 1:** Primary drill pedicle perforations (PDPP) and secondary screw pedicle perforations (SSPP)

Skill level	Group	PDPP	SSPP		
			6 mm	7 mm	8 mm
Expert	FH	0	2	3	8
	AR	0	3	3	6
	*p*	–	0.321	0.325	0.196
Novice	FH	3	18	20	22
	AR	1	7	10	15
	*p*	0.615	0.006	0.013	0.053

The average SSPP for 6-mm screws were 2 perforations (5%) in the FH group and 3 perforations (15%) in the AR group for experts. For 7-mm screws, 3 perforations (15%) occurred in both groups, and for 8-mm screws, the SSPP were 8 perforations (20%) and 6 perforations (15%) in the FH and AR groups, respectively.

For novices (Table 1), the average SSPP for 6-mm screws were 18 perforations (45%) in the FH and 7 perforations (17.5%) in the AR set screws. The difference between the groups was significant (*p* = 0.006). For 7-mm screws, the difference between the FH and AR set screws was 20 perforations (50%) versus 10 (25%) perforation, respectively (*p* = 0.013). For 8-mm screws, the SSPP were 22 perforations (55 %) in the FH and 15 perforations (37.5 %) in the AR set screws, respectively (*p* = 0.053).

**Fig. 7:**
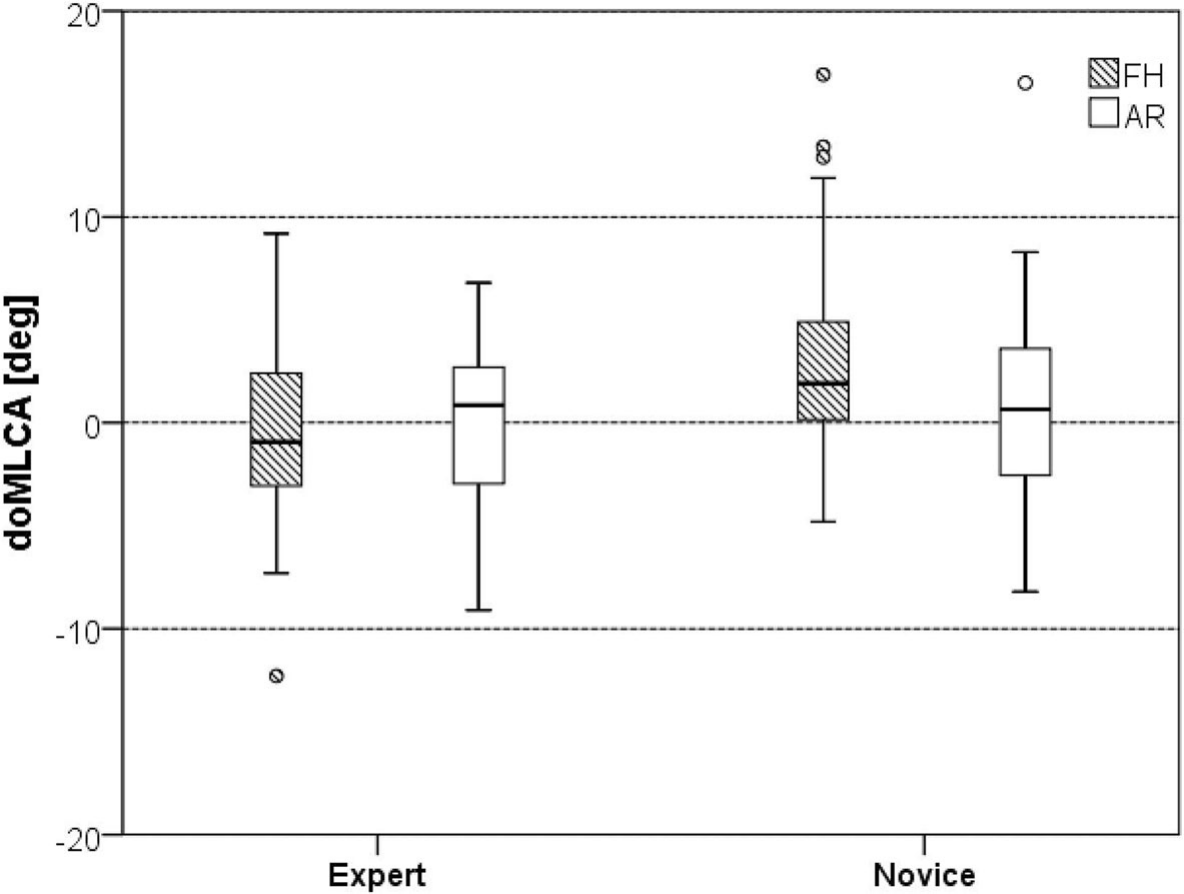
Deviation of the optimal medio-lateral convergence angle (doMLCA)

The average doMLCA was − 0.51° ± 4.20° and 0.14° ± 3.98° in the FH and AR set screws, respectively, for experts (*p* = 0.489) (Fig. 7). In novices, the average doMLCA was 3.23° ± 4.90° and 0.62° ± 4.56° for the FH and AR set screws (*p* = 0.017), respectively.

**Fig. 8:**
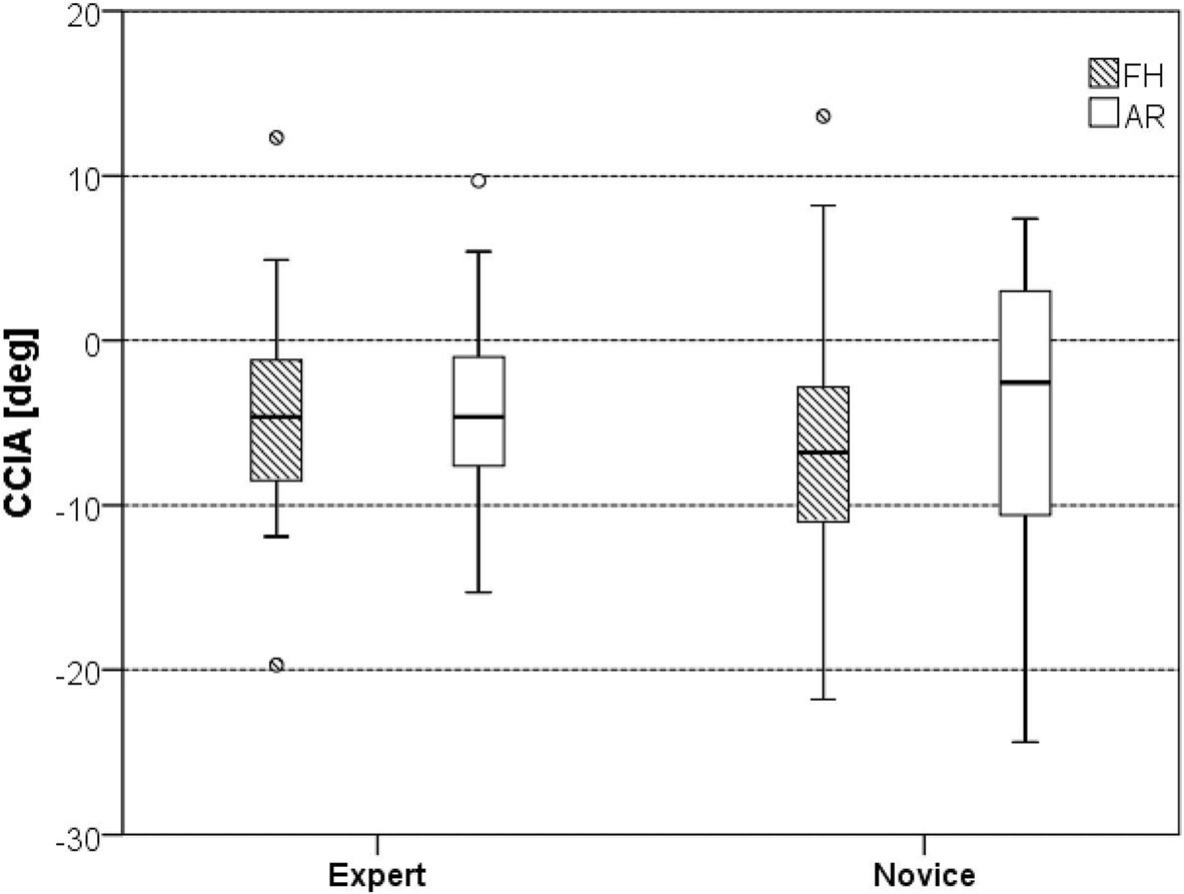
Cranio-caudal inclination angle (CCIA)

There were no significant differences of the CCIA between the AR and the FH set screws nor in the hands of experts nor novices, respectively (Fig. 8). Interestingly, the mean CCIAs were negative for experts and novices in both AR and FH groups, showing that pilot holes were drilled more cranially directed towards the endplate. CCIAs were also categorized in high-, medium-, and low-precision screws as illustrated in Table 2.

**Table 2:** Cranio-caudal inclination angle (CCIA), categorized into low-, medium-, and high-precision

Skill Level	Group	Precision category			*p* value
		High, − 2.5 to + 10°	Medium, − 5 to − 2.5°/+ 10 to + 20°	Low < − 5°/> + 20°	
Expert	FH	12	8	20	0.917
	AR	13	9	18	
Novice	FH	9	4	27	0.040
	AR	20	2	18	

Novices drilled with a higher precision with respect to the CCIA category (*p* = 0.04) with AR. The number of low- and medium-precision screws decreased (27 to 18 and 4 to 2, respectively) towards an increase high-precision category (from 9 to 20).

## Discussion

Correct placement of pedicle screws in spinal instrumentation is critical to provide sufficient screw purchase and avoid injury to neurovascular structures. Reported rates of screw misplacement vary greatly in the literature and range as high as 40% [21]. Several techniques have been developed to improve the precision of pedicle screw placement. The aim of this study was to evaluate the feasibility and precision of AR-navigated pilot hole drilling for pedicle screw placement and to compare placement precision between novices and experienced spine surgeons.

For novice surgeons not experienced in spine surgery, the results of this study showed a significant decrease of primary and secondary screw perforation and an increased precision of cranio-caudal inclination angle and of the optimal medio-lateral convergence in the AR group compared to the traditional free-hand technique. Different modalities for improvement in overall surgical outcomes and training of complex surgical procedures are currently available. These include two-dimensional (2D) fluoroscopic and three-dimensional (3D) computed tomography (CT) renderings to guide operative approach in the perioperative setting. Augmented reality has already been used as an effective tool for training and skill assessment of surgical residents [22]. Compared to virtual reality (VR) simulators, where the whole simulation takes part in a computer graphics (CG) environment, the main advantage of AR simulators is the ability to combine real-life objects with CG images [17].

However, in this study, the AR and FH techniques showed no significant difference in terms of primary or secondary screw perforation, average MAPW, average doMLCA, or cranio-caudal inclination angle in the hands of the expert surgeons. Several studies demonstrated significantly higher accuracy of pedicle screw placement using other navigation systems providing three-dimensional information to the surgeon compared to the free-hand technique [20, 23, 24]. Three-dimensional navigation proved to be of use not only in patients with but particularly when treating spinal deformity [25, 26]. Although similar clinical results are currently missing, AR-based navigation techniques already demonstrated remarkable precision in an experimental setting [27]. As a result of different approaches of measuring precision and accuracy, an exact comparison of specific studies is not useful.

However, projecting the required information directly into the line of sight of the surgeon is considered the natural progression of these well-established methods mitigating the errors associated with attention shift by directly projecting the navigation guidance onto the surgical field [27, 28].

In this study, the surgical procedure was simulated in a laboratory setting using third lumbar sawbone models placed apart from each other without soft tissue covering the posterior structures of the vertebra. We hypothesize that the simplicity of the model used in this study might be one contributing factor explaining why the additional three-dimensional information provided by the AR headset did not influence the precision of screw placement in the expert group. The simplicity of the experimental setup however allowed elimination of potential biases such as variation in anatomy or bone quality.

This study has limitations. The AR navigation technique per se demonstrated technical challenges that still need to be overcome. The exact manual overlay of the 3D model with the real environment, also known as registration, and its correction in case of drift, is still time-consuming. The pronounced bony landmarks were medio-lateral and less so cranio-caudal, making latter alignment of the 3D model with the real anatomy more difficult. In a real operating room setting, the described registration approach would be impractical. Various methods of registration have been described including ultrasound-based techniques, reflective markers mounted on spinous processes, and non-invasive skin placed markers [27–29]. However, further research is currently being undertaken to facilitate automation of the process, and with advancements of technology in the future, the use of dedicated markers might be obsolete.

However, in this study, surgeons were able to perform the manual registration as previously described and the drilling of 40 pedicles in 40 different vertebras in approximately 60–90 min.

Also, this study focused on superimposition of anatomy, while the surgeon had to imagine the optimal trajectory. For the future, adding a virtual representation of the optimal drilling axis could further improve usability and precision.

## Conclusion

In this study, the additional anatomical information provided by the AR headset superimposed to real-world anatomy improved the precision of drilling pilot holes for pedicle screws in a laboratory setting for surgeons not experienced in spine surgery. Further technical development and validations studies are currently being performed to investigate potential clinical benefits of the herein described AR-based navigation approach.

## Data Availability

All data generated or analyzed during this study are included in this published article.

## Abbreviations

AR: Augmented reality
CCIA: Cranio-caudal inclination angle
doMLCA: Deviation of the optimal medio-lateral convergence angle
FH: Free-hand
MAPW: Minimal distance of the drill axis to the pedicle wall
oMLCA: Optimal medio-lateral convergent angle
PDPP: Primary drill pedicle perforations
SSPP: secondary screw pedicle perforations
tMLCA: True medio-lateral convergence angle
